# Ultrasonography of the late-stage knee osteoarthritis prior to total knee arthroplasty: comparison of the ultrasonographic, radiographic and intra-operative findings

**DOI:** 10.1038/s41598-018-35824-3

**Published:** 2018-12-10

**Authors:** Mika T. Nevalainen, Kyösti Kauppinen, Juho Pylväläinen, Konsta Pamilo, Maija Pesola, Marianne Haapea, Juhani Koski, Simo Saarakkala

**Affiliations:** 1 Department of Diagnostic Radiology, Oulu University Hospital, P.O. Box 50, 90029 Oulu, Finland; 2 Medical Research Center Oulu, University of Oulu, P.O. Box 8000, Oulu, Finland; 3 Research Unit of Medical Imaging, Physics and Technology, Faculty of Medicine, University of Oulu, POB 5000, FI-90014 Oulu, Finland; 4 Department of Radiology, Central Finland Central Hospital, Keskussairaalantie 19, 40620 Jyväskylä, Finland; 5 Department of Radiology, Helsinki University Hospital, P.O. Box 100, 00029 Helsinki, HUS Finland; 6 Department of Surgery, Central Finland Central Hospital, Keskussairaalantie 19, 40620 Jyväskylä, Finland; 7 Department of Internal Medicine, Mikkeli Central Hospital, Porrassalmenkatu 35, 50100 Mikkeli, Finland

## Abstract

The purpose of this study was to assess the effectiveness of the ultrasonography (US) on detecting osteoarthritis of the knee, and compare US and radiographic findings to intraoperative total knee arthroplasty (TKA) findings. Fifty-seven late-stage osteoarthritic knees undergoing TKA were evaluated with US and radiography. Standard knee US assessing femoral cartilage damage, osteophytes, effusion, synovitis, and meniscal extrusion was performed. On radiographs, osteophytes, joint space narrowing, and Kellgren-Lawrence grade were evaluated. Corresponding intra-operative findings were assessed during TKA as the gold standard. On the damage of the medial femoral condyle cartilage, the sensitivity of US was high (92%), whereas on the lateral condyle and sulcus area, sensitivities were 58% and 46%, respectively. On osteophytes, the detection rate of the US was remarkable especially on the medial side yielding sensitivities of 90–95%. The sensitivities for detecting effusion and synovitis were also excellent (97%). US detection rate of femoral cartilage damage was in concordance with the radiographic joint space narrowing. For the detection of osteophytes, US provided superior results to radiography particularly on the medial side. In conclusion, US can reliably assess the late-stage OA changes of the knee especially on the medial side of the knee joint.

## Introduction

The prevalence of osteoarthritis (OA) is soaring worldwide with increased age and obesity; currently, the global prevalence is estimated to be 5%^1^. As the knee OA is a common entity with tremendous sosioeconomical impact with 10–13% patients over 60 years or older suffering from it in the USA^1,2^, it is of utmost importance to find precise and quick imaging techniques to detect osteoarthritic changes. Ultrasonography (US) of the knee joint is an emerging imaging technique to evaluate the knee OA. To date, clinical examination followed by the knee radiography have been deemed the gold standard for the diagnosis of the OA, but during the last decade the utility of the US on evaluating the knee joint has been studied vigorously^3,4^. As the radiography offers information preferably on the bone structures, US can be applied to assess the effusion, synovitis, osteophytes, menisci and the femoral cartilage of the knee joint. Moreover, it has been shown that US detects osteophytes more readily than radiographs and that the cartilage defects of the medial femoral condyle correlate well with joint-space narrowing seen on radiographs^5–8^. Significant correlation between US-detected and radiographically-detected osteophytes has been also reported^8–10^. Furthermore, excellent inter-observer agreement has been observed for evaluating the osteoarthritic changes of the knee^11–13^. Also, significant association with pain intensity and clinical symptoms has been found^8,11,13–15^. The diagnostic performance of US on detecting osteoarthritic changes has been compared with magnetic resonance imaging (MRI) and arthroscopy^16,17^: Podlipska *et al*. reported that osteophytes, cartilage changes in the medial femoral condyle and medial meniscal extrusion can be reliably assessed by US as compared to MRI findings^16^. Additionally, Saarakkala *et al*. found that positive findings on US are a strong indicator of arthroscopic degenerative changes, but negative findings do not rule out osteoarthritic changes^17^. Despite the previous studies, it is still unknown whether the actual weight-bearing joint spaces are visualized on US and how plausible the US findings are. The purpose of this study was to assess US findings on patients with late-stage knee OA undergoing total knee arthroplasty (TKA) and compare US findings with radiographic and intra-operative findings.

## Methods

### Patients

Fifty-seven patients scheduled for TKA for late-stage OA of the knee were enrolled consecutively in this study during October 2016 and February 2017. Late-stage OA was defined as eligibility for TKA i.e. a combination of typical clinical history and findings for knee OA supplemented with knee radiographs. Written informed consent was obtained from every patient. The mean patient age was 70 years (range 47 to 84) and 28% were males. The study was carried out in accordance with the Declaration of Helsinki and approved by the Ethical Committee of Central Finland Health Care District, Central Finland Hospital (number 6U/2016).

### Imaging technique and analysis

#### Ultrasonography

US imaging was conducted using the GE LOGIC E9 ultrasound device (GE Healthcare, Milwaukee, WI, USA) with 15 MHz linear transducer (type ML6–15). B-mode imaging settings were kept constant for each subject and the focus was set at the level of region of interest. US of the knee was performed by a single radiologist (M.N.) with expertise on musculoskeletal US. The radiologist conducted the acquisition and analysis for the US evaluation, and was blinded to the clinical and radiographic findings. Conventional US technique was applied to assess the knee joint as described previously^16,17^. First, the knee was scanned with patient in supine position with knee fully extended to evaluate the osteophytes on medial and lateral joint space and the extrusion of the medial and lateral meniscus. Subsequently, the knee was flexed 30° to assess the effusion and synovitis. Doppler imaging was not utilized in this study. Eventually, the knee was flexed as much as possible – typically 90° to 120° – to evaluate the cartilage to femoral sulcus, medial and lateral femoral condyles. The presence and size of osteophytes were evaluated in medial-femoral, medial-tibial, lateral-femoral and lateral-tibial bone margin as follows: Grade 0 = no osteophyte, Grade 1 = marginal/minimal osteophyte, Grade 2 = medium osteophyte and Grade 3 = large osteophyte^5^. Meniscal extrusion was measured as a perpendicular distance (mm) between the most distant meniscus border and line connecting the femoral and tibial bone ends (measuring below osteophytes if present) and over 4 mm was defined as a sign of extrusion^7^. Effusion was defined as at least 4 mm thickness of fluid in suprapatellar pouch^4^. Moreover, synovitis was defined as heterogenous synovial proliferation of at least of over 4 mm of thickness in the suprapatellar pouch and parapatellar recesses^4^. The femoral cartilage was graded as follows: Grade 0 = normal (a monotonous anechoic band having a sharp hyperechoic anterior and posterior interfaces), Grade 1 = loss of the normal sharpness of cartilage interfaces and/or increased echogenicity of the cartilage, Grade 2 A = in addition to above changes, clear local thinning (less than 50%) of the cartilage, Grade 2B = local thinning (more than 50% but less than 100%) and Grade 3 = total loss of the cartilage^17^.

#### Radiography

All patients underwent bilateral weight-bearing postero-anterior radiography on the same day as the US examination. The X-ray beam was 10° caudally angulated and the knee was supported by a frame in 20° flexion and foot in 5° external rotation. The knees were assessed by the same radiologist for osteophytes, joint space narrowing and Kellgren-Lawrence grades^18^. Osteophytes were graded in medial-femoral, medial-tibial, lateral-femoral and lateral-tibial bone margin as follows: Grade 0 = no osteophyte, Grade 1 = marginal/minimal osteophyte, Grade 2 = a definite osteophyte. Joint spaces (medial and lateral separately) were defined either normal or narrowed. Ultimately, the total Kellgren-Lawrence grade was given for both the medial and the lateral compartment of the knee joint. The reader (same radiologist performing US assessment, M.N.) was blinded to clinical and US findings.

### Total knee arthroplasty findings

The TKA operation was performed on average 67 days (range 2 to 181 days) after the US evaluations by 3–5 orthopedic surgeons with at least 10 years of TKA experience. The surgeons were blinded to the US findings, but not to clinical history and radiography findings. The routine TKA protocol was performed using medial parapatellar approach, and the surgical findings were collected as follows: wearing of the cartilage on the femoral sulcus, medial and lateral condyle (normal, marked softening, distinct wearing), osteophytes at medial-femoral, medial-tibial, lateral-femoral and lateral-tibial (yes, no), meniscal extrusion or maseration (yes, no), clinically seen effusion (yes, no) and marked synovial proliferation (yes, no). The grading was kept simple due to several different surgeons performing the TKAs.

### Statistical analysis

For statistical analyses, cut-offs were applied to create dichotomous score on certain variables: US-detected cartilage damage was categorized as non-significant (Grades 0 and 1) or significant (Grades 2 A, 2B and 3); US-detected osteophytes as non-significant (Grades 0 and 1) or significant (Grades 2 and 3); radiographically detected osteophytes as non-significant (Grades 0 and 1) or significant (Grade 2); and intraoperative cartilage damage as non-significant (normal) or significant (marked softening, distinct wearing). Data of US and radiography findings are given as numbers of true positive and negative findings according to intraoperative findings. Sensitivity, specificity, accuracy, positive predictive value, and positive and negative likelihood ratios with their 95% confidence intervals were calculated for each finding. The confidence intervals for the first four were calculated using Wilson score method without continuity correction^19^ and log method^20^ for the last two. The sensitivities between US and radiography were compared within positive intraoperative findings using Mc-Nemar’s test. P-value < 0.05 was considered statistically significant. SPSS 24.0 was used in analyzing the data.

## Results

### US versus TKA findings

**Figure 1:**
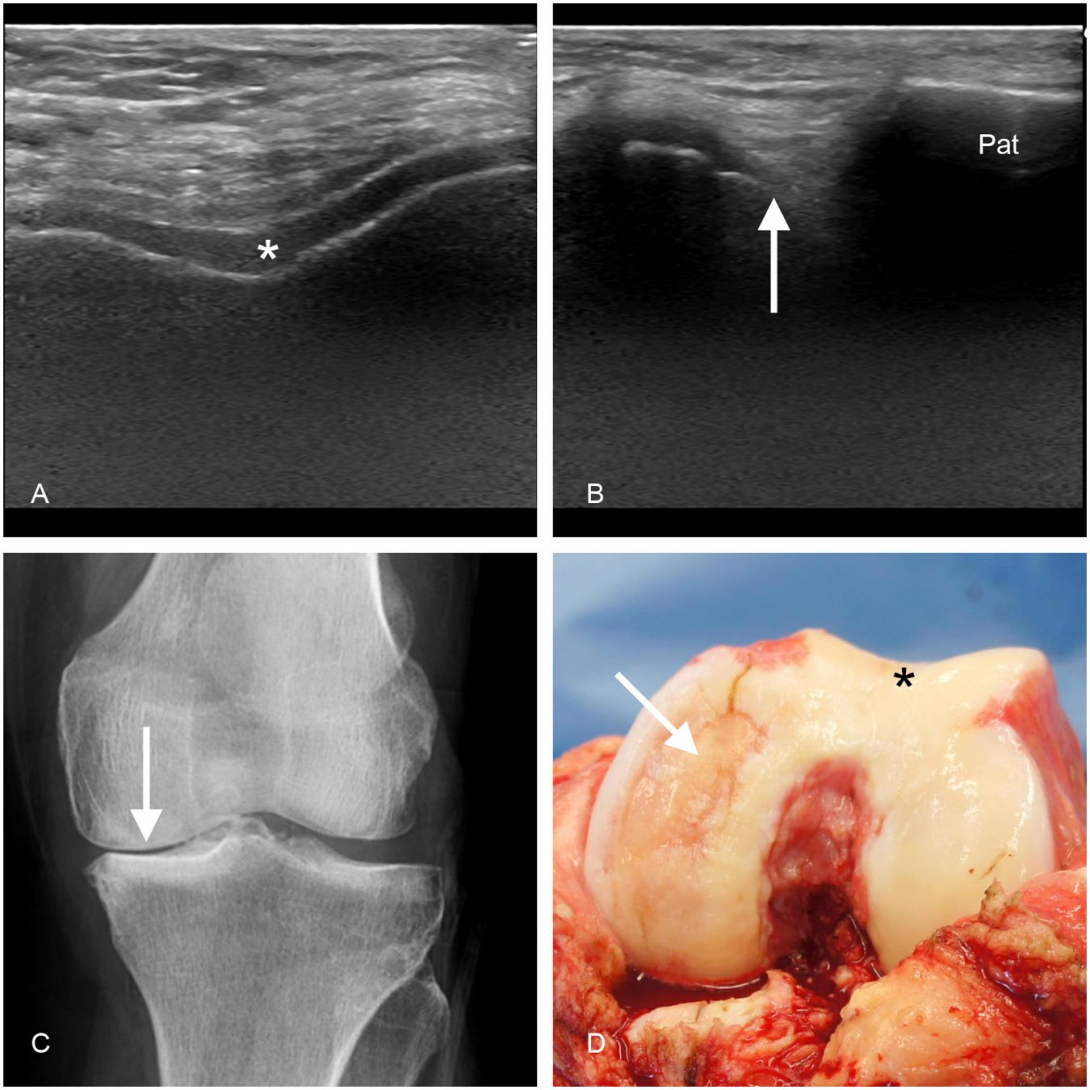
The cartilage view on ultrasonography (US), on radiography and during total knee arthroplasty (TKA). On femoral sulcus, axial US-view demonstrates a normal cartilage (white asterisk) without damage (**A**). On medial femoral condyle, axial parapatellar US-view (patella on the right side, marked as Pat) shows a distinct damage (arrow) on the cartilage (**B**). The corresponding radiography depicts a clear narrowing (arrow) of the medial joint space (**C**). Ultimately, the photography of femoral cartilage surfaces taken during the TKA reveals intact femoral sulcus (asterisk) and full-thickness cartilage damage of the medial femoral condyle (arrow) (**D**).

When comparing the US findings with the intraoperative findings on the 57 knees that underwent TKA, the US examination performed well. For the cartilage degeneration at the femoral medial condyle the sensitivity of the US was 92% and specificity was 50%; moreover, the accuracy was 88% and the positive predictive value was 94%. For the lateral condyle and for the sulcus area, the sensitivities were 58% and 46%; the specificities were 76% and 84%; accuracies were 70% and 67%; and the positive predictive values were 55% and 71%, respectively. Figure 1 depicts an example of the cartilage view on US, radiography and TKA. Concerning the evaluation of the osteophytes, the detection rate of the US was outstanding especially on the medial side: For the femoral medial condyle the sensitivity, specificity, accuracy and positive predictive value were 95%, 50%, 93% and 98%, respectively. For the femoral lateral condyle they were 93%, 27%, 75% and 78%, respectively. For the tibial medial condyle the sensitivity, specificity, accuracy and positive predictive value were 90%, 75%, 88% and 96%, respectively. For the tibial lateral condyle the corresponding values were 65%, 76%, 72% and 59%, respectively. The sensitivities for detecting effusion and synovitis were also excellent yielding a sensitivity of 97% and 97%, respectively. For the damage of the medial and lateral meniscus the sensitivities were 93% and 58%, respectively. The positive likelihood ratios for US findings varied between 1.02 and 3.59, and the negative likelihood ratios between 0.11 and 0.64. Table 1 summarizes the comparison of US and TKA findings.

**Table 1:** Performance of ultrasonography on detecting osteoarthritic changes of the knee when using the intra-operative findings of total knee arthroplasty as a gold standard.

	TP/N1	Sensitivity, % (95% CI)	TN/N2	Specificity, % (95% CI)	N3	Accuracy, % (95% CI)	Positive predictive value, % (95% CI)	Positive likelihood ratio (95% CI)	Negative likelihood ratio (95% CI)
Wearing of the cartilage									
Femoral medial condyle	47/51	92 (81–97)	3/6	50 (19–81)	50	88 (77–94)	94 (84–98)	1.84 (0.82, 4.12)	0.16 (0.05, 0.54)
Femoral lateral condyle	11/19	58 (36–77)	29/38	76 (61–87)	40	70 (57–80)	55 (34–74)	2.44 (1.23, 4.86)	0.55 (0.32, 0.96)
Sulcus	12/26	46 (29–65)	26/31	84 (67–93)	38	67 (54–78)	71 (47–87)	2.86 (1.16, 7.06)	0.64 (0.44, 0.95)
Osteophytes									
Femoral medial condyle	52/55	95 (85–98)	1/2	50 (9–91)	53	93 (83–97)	98 (90–100)	1.89 (0.47, 7.57)	0.11 (0.02, 0.64)
Femoral lateral condyle	39/42	93 (81–98)	4/15	27 (11–52)	43	75 (63–85)	78 (65–87)	1.27 (0.92, 1.74)	0.27 (0.07, 1.06)
Tibial medial condyle	44/49	90 (78–96)	6/8	75 (41–93)	50	88 (77–94)	96 (85–99)	3.59 (1.08, 11.97)	0.14 (0.05, 0.34)
Tibial lateral condyle	13/20	65 (43–82)	28/37	76 (60–87)	41	72 (59–82)	59 (39–77)	2.67 (1.39, 5.13)	0.46 (0.25, 0.86)
Effusion	28/29	97 (83–99)	3/28	11 (4–27)	31	54 (42–67)	53 (40–66)	1.08 (0.93, 1.25)	0.32 (0.04, 2.91)
Synovitis	35/36	97 (86–100)	1/21	5 (1–23)	36	63 (50–74)	64 (50–75)	1.02 (0.91, 1.14)	0.58 (0.04, 8.85)
Meniscus									
Femoral medial condyle	42/45	93 (82–98)	3/12	25 (9–53)	45	79 (67–88)	82 (70–90)	1.24 (0.89, 1.74)	0.27 (0.06, 1.16)
Femoral lateral condyle	14/24	58 (39–76)	24/32	75 (58–87)	38	68 (55–79)	64 (43–80)	2.33 (1.17, 4.65)	0.56 (0.33, 0.93)

TP/N1 = Number of true positives / positive intraoperative findings. TN/N2 = Number of true negatives / negative intraoperative findings.

N3 = Total number of readings concordant with intraoperative findings in 57 knees.

95% CI = 95% confidence interval.

### US versus radiography

**Figure 2:**
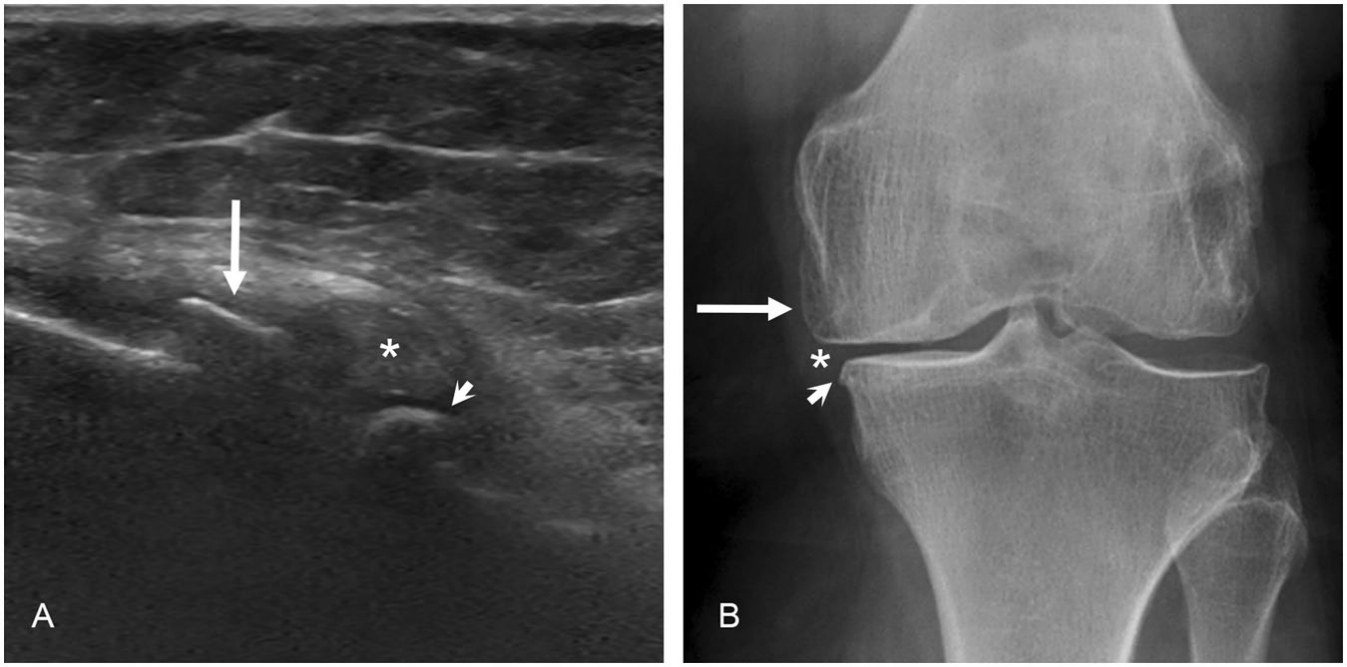
The ultrasonography (US) and radiography of the medial compartment of the knee. (**A**) US image shows a significant osteophyte on the medial-femoral (arrow) and medial-tibial (arrowhead) sites. (**B**) On the corresponding radiography, no corresponding osteophyte is detected on the medial-femoral site (arrow), whereas only medium osteophyte is seen on the medial-tibial site (arrowhead). The asterisks describe the dislocation of the medial meniscus to the joint space.

When comparing US with radiography – using the TKA findings as the gold standard – the detection rate of cartilage damage was in line with the radiographic joint space narrowing findings: For the medial joint space, the sensitivities of the US versus radiography were 92% and 92%, the specificities 50% and 67%, the accuracies 88% and 89%, and the positive predictive values were 94% and 96%, respectively. For the lateral joint space, the US versus radiography sensitivities were 58% and 42%, specificities 76% and 82%, accuracies 70% and 68%, and the positive predictive values were 55% and 53%. For the detection of osteophytes, the US yielded superior results than the radiography especially on the medial side (Fig. 2): At the femoral medial condyle, the US versus radiography sensitivities were 95% and 44%; at the femoral lateral condyle 93% and 24%; at the tibial medial condyle 90% and 76%; and at the tibial lateral condyle 65% and 70%. Table 2 summarizes the radiography versus intraoperative statistics. Finally, Table 3 demonstrates diagnostic efficiencies of the US and radiography when using the TKA findings as the gold standard.

**Table 2:** Performance of radiography on detecting osteoarthritic changes of the knee when using the intra-operative findings of total knee arthroplasty as a gold standard.

	TP/N1	Sensitivity, % (95% CI)	TN/N2	Specificity, % (95% CI)	N3	Accuracy, % (95% CI)	Positive predictive value, % (95% CI)	Positive likelihood ratio (95% CI)	Negative likelihood ratio (95% CI)
Joint space narrowing									
Femoral medial condyle	47/51	92 (81–97)	4/6	67 (30–90)	51	89 (79–95)	96 (86–99)	2.76 (0.89, 8.60)	0.12 (0.04, 0.35)
Lateral medial condyle	8/19	42 (23–64)	31/38	82 (67–91)	39	68 (56–79)	53 (30–75)	2.29 (0.98, 5.36)	0.71 (0.47, 1.07)
Osteophytes									
Femoral medial condyle	24/55	44 (31–57)	2/2	100 (34–100)	26	46 (33–58)	100 (86–100)	2.63 (0.21, 33.5)^a^	0.56 (0.45, 0.71)
Femoral lateral condyle	10/42	24 (13–39)	13/15	87 (62–96)	23	40 (29–53)	83 (55–95)	1.79 (0.44, 7.23)	0.88 (0.68, 1.14)
Tibial medial condyle	37/49	76 (62–85)	5/8	63 (31–86)	42	74 (61–83)	93 (80–97)	2.01 (0.81, 5.00)	0.39 (0.19, 0.81)
Tibial lateral condyle	14/20	70 (48–85)	28/37	76 (60–87)	42	74 (61–83)	61 (41–78)	2.88 (1.52, 5.44)	0.40 (0.20, 0.79)
KL-grading									
Medial	46/51	90 (79–96)	4/6	67 (30–90)	50	88 (77–94)	96 (86–99)	2.71 (0.87, 8.42)	0.15 (0.05, 0.40)
Lateral	15 (19)	79 (57–91)	26/38	68 (53–81)	41	72 (59–82)	56 (37–72)	2.50 (1.48, 4.22)	0.31 (0.13, 0.75)

TP/N1 = Number of true positives / positive intraoperative findings. TN/N2 = Number of true negatives / negative intraoperative findings.

N3 = Total number of readings concordant with intraoperative findings in 57 knees.

95% CI = 95% confidence interval.

^a^Calculated after adding 0.5 to the counts in all four cells of the observed table as suggested by Altman (2000) due to no false positives.

**Table 3:** Ultrasonography (US) findings versus radiography findings when intra-operative total knee arthroplasty findings were used as a gold standard.

US finding vs. radiography finding	Total	US+/R+ N (%)	US+/R− N (%)	US−/R+ N (%)	US−/R− N (%)	P
Wearing of the cartilage vs. joint space narrowing						
Femoral medial condyle	51	45 (88.2)	2 (3.9)	2 (3.9)	2 (3.9)	>0.999
Femoral lateral condyle	19	7 (36.8)	4 (21.1)	1 (5.3)	7 (36.8)	0.375
Wearing of the cartilage vs.KL-grading						
Femoral medial condyle	51	44 (86.3)	3 (5.9)	2 (3.9)	2 (3.9)	>0.999
Femoral lateral condyle	19	9 (47.4)	2 (10.5)	6 (31.6)	2 (10.5)	0.289
Osteophytes						
Femoral medial condyle	55	22 (40.0)	30 (54.5)	2 (3.6)	1 (1.8)	<0.001
Femoral lateral condyle	42	10 (23.8)	29 (69.0)	0 (0.0)	3 (7.1)	<0.001
Tibial medial condyle	49	32 (65.3)	12 (24.5)	5 (10.2)	0 (0.0)	0.143
Tibial lateral condyle	20	9 (45.0)	4 (20.0)	5 (25.0)	2 (10.0)	>0.999

US+ = positive in ultrasound, US− = negative in ultrasound, R+ = positive in radiography, R− = negative in radiography.

## Discussion

In this study, we compared the US findings of the late-stage knee OA with the radiographic and intra-operative findings in the TKA procedure. To the best of our knowledge, this is the first study to deploy direct visualization of the knee joint by using TKA as the gold standard for US and radiographic findings. Previously, studies by Saarakkala *et al*.^17^ and Koski *et al*.^5^ have applied arthroscopy as the gold standard; Saarakkala’s team found that correlation of severity of cartilage damage between US and arthroscopy varied from insignificant to significant depending on the site: at the sulcus area the correlation was highest and at the medial condyle also significant, but at the lateral condyle insignificant. They concluded that a positive finding on US is a potent indicator of arthroscopic degenerative changes of cartilage, but a negative finding does not rule out degenerative changes^17^. Subsequently, Koski *et al*. showed a significant correlation between US detected osteophytes and the degenerative cartilage changes at arthroscopy at the medial compartment^5^. However, neither of these studies comprehensively compared all the US findings (effusion, synovitis, osteophytes, cartilage damage and meniscal pathology) to the macroscopic intra-operative findings; for this purpose, the direct visualization of the joint during TKA indubitably offers the best gold standard. In concordance with previous studies^5,17^, our study confirms that US performs better on the medial compartment of the knee. Using the MRI as a gold standard, Podlipska *et al*. reported that the ability of US to detect medial cartilage damage was good, whereas for the lateral femoral condyle it was only fair^16^. The superior performance at the medial aspect of the knee may be due to the better acoustic window than on the lateral side. Moreover, the bony contour is usually more explicit on the medial edge.

With regard to detection of osteophytes, this is the first study to compare US and radiographic findings to the actual macroscopic findings. Here, US outranked radiography in every compartment (medial-femoral, lateral-femoral, medial-tibial and lateral-tibial) when TKA findings were used as a gold standard, but statistically significant difference was seen only on femoral osteophytes. Furthermore, likelihood ratios were interpreted as described by McGee (2002): Positive and negative likelihood ratios show how the probability of the OA changes when the finding is present or absent, respectively. For instance, positive likelihood ratios of 2.0 and 3.0 increase the probability of OA by 15% and 20%, respectively, whereas negative likelihood ratios of 0.2 and 0.5 decrease the probability of OA by 30% and 15%, respectively. Likelihood ratio of 1 reflects lack of diagnostic value. However, it should be reminded that likelihood ratios are calculated using logarithms (i.e. they are not linear) and therefore meticulous interpretation is recommended^21^. Results similar to ours have been also shown by Podlipska *et al*., who found the diagnostic performance of US to detect any osteophytes in the medial and lateral femur and tibia was excellent to good when using MRI as a gold standard^16^. Moreover, Koski *et al*. stated that US detected more osteophytes than radiography at both the medial (65% vs. 48%) and lateral compartments (70% vs. 60%); significant statistical correlation was found between US and radiography at the medial side, but only low correlation at the lateral side^5^. Taken together, our results confirm the recent studies suggesting that US is more sensitive in the detection of osteophytes than radiography^5,6,8,16^. Previously, good correlation between US findings and radiographic severity (Kellgren Lawrence grade) has been demonstrated^8–10^ with preference on the medial aspect of the knee^6^. Our results here again confirm this as US-detected cartilage damage correlated well with radiographically-detected joint-space narrowing and Kellgren Lawrence grade.

There are some limitations in this study. First, the high number of osteoarthritic findings – the patients representing late-stage knee OA scheduled for TKA – creates bias to this study. This reflects mostly as the low specificity obtained by the US examination as almost every patient had a positive finding. However, this could not have been avoided, since we wanted to use the direct visualization of the knee joint during TKA as the ultimate gold standard here. Second, the time from the US examination to the TKA operation varied and therefore the inflammatory synovial changes and especially the effusion could have changed. Third, the flexion angle of the knee was not standardized leading to better visualization of the femoral cartilage on some patients; however sufficient acoustic window with at least 90° flexion was obtained with every patient. Fourth, the high BMI of few patients weakened the diagnostic US window. Finally, the relatively large number of operating orthopedic surgeons induced variation to the classification of the TKA findings; accordingly, the intra-operative grading was kept as simple and explicit as possible. Moreover, the surgeons were not blinded to the radiography findings, which could have created bias on the intra-operative classification.

In conclusion, US can be used reliably to evaluate the late-stage OA changes of the knee particularly on the medial side. Our study shows outstanding sensitivities for effusion, synovitis, osteophytes, cartilage damage and meniscal pathology. Moreover, US yields superior detection of osteoarthritic changes as compared to radiographs.

## Data Availability

The datasets generated during and analyzed during the current study are available from the corresponding author on reasonable request.
